# Identification and Targeting of Stem Cell-Activated Pathways in Cancer Therapy

**DOI:** 10.1155/2019/8549020

**Published:** 2019-06-04

**Authors:** Alfredo Budillon, Steven Curley, Roberta Fusco, Rita Mancini

**Affiliations:** ^1^Istituto Nazionale Tumori-IRCCS-Fondazione G. Pascale, 80121 Napoli, Italy; ^2^Surgical Oncology, CHRISTUS Trinity Mother Frances Hospital Tyler, Texas, USA; ^3^Department of Clinical and Molecular Medicine, Sapienza University of Rome, 00161 Rome, Italy

The hierarchical organization and heterogeneity that are present within malignancies have been recently attributed to stem cell-like subset of tumor cells. Cancer Stem Cells (CSCs) have gained an exclusive interest as they are identified to push tumor growth and seed metastasis and are responsible for therapy failure and tumor resistance [1].

The molecular mechanism that drives the CSC population has started to emerge, and the identification and origin of factors that maintain or even induce a CSC phenotype remain an intense area of research. This special issue contains seven articles, four reviews, and three original studies, highlighting the recent advances in CSC-activated pathways, with particular emphasis on the cross-talk between the CSC and the tumor microenvironment (TME). The studies presented here also highlighted compounds (e.g., epi-drugs) that are described to modulate CSCs and TME-activated pathways and thus can be subsequently exploited for therapeutic use.

P. Gener et al., in a review article, discussed the overlapping phenotype between CSC and mesenchymal cancer cells, in terms of origin, activated pathways, and the implication for cancer treatment. Indeed, similarly to CSC, a link between epithelial to mesenchymal transition of cancer cells and metastasis as well as resistance to anticancer agents has been proposed [2]. Although other reports suggested that EMT is not necessary for metastasis, but rather it is the tumor microenvironment that regulate epithelial or mesenchymal state, still agent able to target common pathways regulating both CSC and EMT (e.g., TGF-*β* and NF-*κβ* signaling) showed antimetastatic potential and improved anticancer treatments. However, the authors also suggested that the strategies to prevent tumor remission by targeting the highlighted pathways should include integrated combining approach that take in account the intrinsic dynamism characterizing, within the tumor, the interconversion capacity of non-CSCs to new CSCs and mesenchymal cells, via EMT activation.

As suggested above, several evidences demonstrated a critical role of the microenvironment in regulating CSC and their involvement in tumor progression. In this regard C. Ciardiello et al. reviewed the bidirectional communication mechanisms between the CSC and the microenvironment, mediated by extracellular vesicles (EVs). EVs are considered as one of the most effective vehicles of information among cells, and recent findings demonstrated that they play an important role in cancer development and progression, by transferring information between cancer cells as well as between cancer cells and tumor microenvironment, at both paracrine and systemic level [3]. EVs are highly heterogeneous; however, they can be classified in two major classes: the shed microvesicles, formed through the direct budding of the plasma membrane, and the exosomes, small size vesicles (30-150 nm) generated through the classical endosome-multivesicular body pathway [3].

The specific role of this latter class of vesicles was reviewed by J. Xu et al. They speculate that exosomes play a role in maintaining homeostasis between non-CSCs and CSCs within the tumor. The authors described how exosomes can regulate both EMT and CSC phenotype activating stem-related signaling pathways (e.g., Wnt, Notch, or Hedgehog pathways) by docking on cancer cell receptors and/or transferring their cargo inside the cells.

Epigenetic alterations play an important role in the initiation and progression of several cancers. Moreover, since epigenetic alterations are dynamic and generally reversible, epigenetic manipulation has emerged as an attractive novel anticancer treatment.

Increasing evidences support the significance of epigenetic regulation in CSC features [4]. DNA methylation and histone acetylation are two epigenetic modifications that participate in the modulation of many gene expressions, which regulate important cellular activity such as proliferation, differentiation, and migration [5]. While the role of DNA methylation in CSCs is relatively well established, the role of histone and nonhistone protein acetylation is still not completely clear [4]. In this context, different hematological and solid tumors are characterized by deregulation of the protein acetylation pattern as a result of genetic or epigenetic changes [6]. Protein acetylation is catalyzed by acetyltransferases and deacetylases, through the addition and removal of acetyl groups to lysine residues, respectively [5]. Small molecule inhibitors of both acetyltransferases and deacetylases are in clinical trials, and four deacetylase inhibitors have been approved by FDA for the treatment of hematological malignancies [6].

In this issue, D. Trisciuoglio et al. reviewed the physiological and pathological roles of acetyltransferase enzymes as well as their involvement in the regulation of stem cell renewal and differentiation in both normal and cancer cell population. The authors also discussed the potential of acetyltransferase inhibitors as novel anticancer drugs.

Two additional epi-drugs, 5-azacytidine (AZA) and 5-aza-2 ′-deoxycytidine (DAC), have been also approved by FDA in hematological tumors, and several studies have shown their therapeutic potential also in solid tumors [5].

Here, K. Agrawal et al. demonstrated for the first time the influence of stromal cells and of their secretome on cancer cell response to AZA and DAC. In details, considering that in metastatic setting the interactions between colorectal cancer cells (CRCs) and stroma at the distant metastatic sites are important, the authors investigated the effects of colon-unassociated normal human foreskin and lung fibroblasts, of their radiation-induced senescent counterparts and of their conditioned medium, on CRC cell response to AZA and DAC. Interestingly, although opposite effects of fibroblasts and of their conditioning medium on AZA and DAC antitumor effects were reported and discussed, the authors suggested the potential of the tumor-stroma ratio in predicting the outcome of DNA-demethylating epigenetic cancer therapy.

Finally, this special issue contains two additional original studies on two interesting topics. The first study by Q.-Y. Liu et al. deals with the potential of human normal mesenchymal stem cells (MSCs). MSCs can be isolated from multiple sources including bone marrow, fat, umbilical cord, and menstrual blood and hold a great potential in regenerative medicine and in cell-based therapies in different clinical applications [7]. Interestingly, the authors presented several evidences, both in vitro and in vivo xenograft model, demonstrating that human menstrual blood-derived stem cells can inhibit cervical cancer cell growth and thus suggesting an original novel cell-therapy anticancer approach.

The second study by F. Basit et al. evaluated the expression of the Myc oncogenes and Mxd antagonists in hematopoietic stem cell and myeloid progenitor populations in the Flt3-ITD-knockin myeloproliferative mouse model. Indeed, mutations in the FLT3 gene have been reported in acute myeloid leukemia (AML) and were associated with poor prognosis, while Myc dysregulation has been reported in hematological malignancies. The authors, by highlighting a cross-talk between the FLT3 receptor and Myc, suggested the potential of combination therapies with tyrosine kinase inhibitors and Myc antagonists in treating AML.
